# Contact-Angle-Guided Semi-Cured Slot-Die Coating Eliminates Air Entrapment in LED Multilayer Films

**DOI:** 10.3390/polym17111436

**Published:** 2025-05-22

**Authors:** Zikeng Fang, Jiaqi Wan, Chenghang Li, Henan Li, Ying Yan

**Affiliations:** 1 State Key Laboratory of High-Performance Precision Manufacturing, Dalian University of Technology, Dalian 116024, China; fangzikeng@mail.dlut.edu.cn (Z.F.); wanjiaqi@mail.dlut.edu.cn (J.W.); 201961166@mail.dlut.edu.cn (H.L.); 2 College of Transportation, Ludong University, Yantai 264025, China; lch@ldu.edu.cn

**Keywords:** LED polymer multilayer films, air entrainment mechanism, interfacial wettability, semi-cured slot-die coating

## Abstract

LED polymer multilayer films offer clear advantages over single-layer coatings, such as minimized particle settling, finer control over particle distribution, and more precise spectral tuning. However, the standard “coat–dry–coat” process for these multilayer systems often traps air bubbles, degrading film quality and uniformity. This study investigates the air entrainment mechanism in multilayer film formation. Bubbles form when the cured bottom layer exhibits a low contact angle, which destabilizes the advancing liquid front. High-speed microscopy captured these interfacial dynamics, and contact-angle measurements quantified the wetting behavior. Numerical simulations further demonstrated that reduced wettability and vortex formation drive air entrainment. To mitigate air entrainment, a semi-cured slot die coating approach was proposed to modify the surface wettability and suppress the flow instabilities. Incorporating temperature-dependent viscosity into the simulation model improved its predictive accuracy, cutting the error in predicted coating-gap limits from 11.49% to 4.99%. This combined strategy delivers reliable, bubble-free multilayer films and paves the way for more consistent, high-quality LED polymer applications.

## 1. Introduction

In recent years, the increasing demand for advanced lighting technologies has driven significant progress in the development of LED polymer multilayer films [1,2]. Compared to conventional single-layer mixed LED films, these multilayer structures offer distinct advantages in terms of both functionality and performance [3]. The LED polymer multilayer films effectively suppress particle sedimentation, thereby bolstering the overall performance and lifespan of LED devices [4,5]. Furthermore, the multilayer design enables precise control over the spatial distribution and interaction of various particles, which facilitates the creation of customized, high-performance lighting systems that meet diverse application requirements [6]. This structural versatility is particularly valuable in modern lighting, where performance parameters such as brightness, uniformity, and chromaticity must be finely tuned for optimal user experience. Another critical advantage of LED polymer multilayer films is their ability to achieve precise spectral control [7,8,9]. Through the careful selection and strategic arrangement of fluorescent particles and encapsulation materials, the emission spectrum can be precisely tailored to meet specific application requirements. This approach not only improves color rendering accuracy and enhances energy efficiency, but also contributes to greater visual comfort across a variety of lighting environments [10,11]. However, despite these promising benefits, the prevailing method used to fabricate LED polymer multilayer films, known as the “coat–dry–coat” method, suffers from several inherent limitations. The defects can limit the performance and reliability of LED devices, underscoring the need for comprehensive investigation and innovative solutions.

Bubble behavior during film fabrication has long been a focal point of both experimental and theoretical research due to its direct impact on coating quality and optical performance [12,13,14]. Air entrainment during the coating process can lead to bubble defects, reduced film uniformity, and degraded optical properties [15,16]. Numerous studies have revealed that bubble entrainment, detachment, and escape are strongly influenced by flow field characteristics, fluid–interface interactions, and material properties such as viscosity and surface tension [17,18].

Recent investigations have expanded the understanding of these phenomena. For instance, Uriarte et al. [19] employed a coupled phase-field and Navier–Stokes approach to evaluate the effects of substrate tilt, contact angle, and external flows, concluding that the contact angle is a critical factor in bubble detachment. In our previous studies [20], finite element modeling was conducted to investigate the mechanisms of bubble entrainment during film preparation. The results indicated that viscous and inertial forces dominate the process. Moreover, the introduction of deflectors was shown to significantly increase the allowable substrate velocity, thereby mitigating bubble formation. Gatapova et al. [21] provided a detailed visualization of bubble formation and spontaneous rupture within liquid films, highlighting the instability-driven mechanisms of bubble dynamics. Meanwhile, Dasouqi et al. [22] used high-speed smoke visualization and stereometric tools to experimentally capture the entire bubble escape process, tracking deformation and coalescence in real-time. To improve film uniformity and reduce defects in LED film production, Oh et al. [23] introduced a vacuum pressure control method to suppress bubble entrainment, showing significant improvements in coating consistency. In addition, Ahmad et al. [24] developed a two-dimensional CFD model using the volume of fluid (VOF) method to study bubble growth and deformation during the fluid-to-solid transition, offering insights into phase-dependent behavior.

At present, research on bubble defects during the coating process has primarily focused on single-layer polymer film fabrication, with particular emphasis on the mechanisms of air entrainment, detachment, and escape under various processing parameters [25,26,27,28]. These studies have laid a theoretical foundation for understanding bubble dynamics in coating systems [29,30]. However, multilayer polymer film structures impose more stringent requirements on film quality and interfacial integrity [31,32]. The presence of bubbles in such multilayer systems leads to more complex defect behaviors, including interfacial delamination and inhomogeneous optical properties. Despite this, studies specifically addressing air entrainment formation and evolution in polymer multilayer films remain limited, highlighting the need for further investigation in this area.

Accordingly, systematic comparisons of air entrainment during both single-layer and multilayer polymer film fabrication processes are conducted to elucidate the mechanisms responsible for bubble defect formation in stacked multilayer film coatings. To address these issues, a semi-cured slot die coating strategy is proposed as an innovative approach to effectively suppressing air entrainment in polymer multilayer film production. Furthermore, a slot die coating air entrainment model incorporating temperature-dependent fluid viscosity is developed. Overall, the findings of this research provide the mechanisms of bubble defect formation in the polymer multilayer film fabrication and offer practical guidance for the high-quality fabrication of defect-free polymer multilayer films.

## 2. Materials and Methods

### 2.1. Materials

In this study, the coating liquid was a colorless and transparent organic silicone gel, KER-2600, supplied by Shin-Etsu Chemical Co., Ltd., Tokyo, Japan. Although this silicone gel is not a standard LED polymer, it shares comparable properties with the standard LED polymer, such as high optical transparency, moderate viscosity, and thermal stability. Moreover, its well-defined curing behavior and ease of experimental handling make it suitable for fundamental mechanism studies. Importantly, the rheological characteristics of the selected silicone gel, including its shear-dependent viscosity and surface tension behavior, are within the same range as those of commonly used LED-grade polymer matrices. While absolute material values may differ slightly from those of the standard LED polymer, the governing fluid mechanical behavior remains the same, making the conclusions of this study broadly applicable to real standard LED polymer formulations.

**Figure 1:**
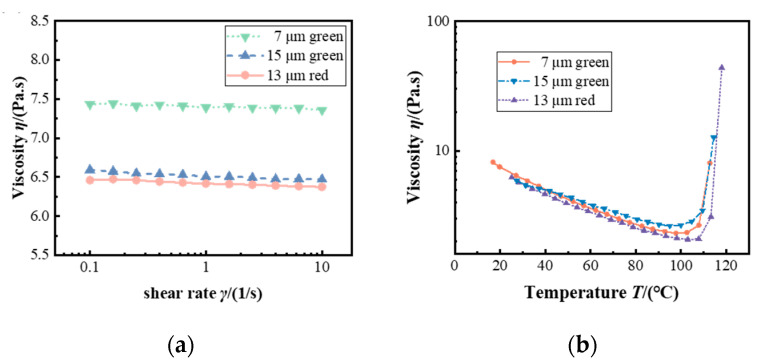
(**a**) Viscosity of different polymer suspensions; (**b**) viscosity of polymer suspensions at different temperatures.

The experimental mixture was prepared by combining two components of KER-2600 in a 1:1 volume ratio, followed by the addition of fluorescent particles (from the Changchun Institute of Applied Chemistry, Chinese Academy of Sciences). To ensure the uniformity of the suspension, a defoaming mixer (ZYMC-350VS, Shenzhen ZYE Science & Technology Co., Ltd., Shenzhen, China) equipped with a vacuum system was used. The mixture was stirred at 2000 rpm under a vacuum pressure of −100 kPa for 15 min. The viscosity of the mixture with a 10% volume concentration of fluorescent particles was measured using a rheometer. Rheological measurements were performed on an AR2000ex rheometer (TA Instruments, New Castle, DE, USA), which provides precise control of the shear rate and temperature for accurate fluid characterization. As shown in Figure 1a, the average viscosities of silica gel mixed with 7 μm and 15 μm green phosphor particles and 13 μm red phosphor particles are 7.4 Pa·s, 6.52 Pa·s, and 6.42 Pa·s, respectively. Figure 1b illustrates the temperature-dependent behavior of viscosity below 110 °C, and the viscosity decreases with increasing temperature. However, a rapid increase in viscosity is observed due to the onset of cross-linking reactions and subsequent solidification of the fluid beyond 110 °C. Detailed experimental parameters of the silicone gel and phosphor particles are summarized in Table 1.

In this study, the silicone gel–phosphor system is used as an alternative model for studying the optical properties of LED polymers. The rationale for this choice is based on two main factors: material property matching and industrial relevance. In terms of material properties, the refractive index of the silicone gel closely matches that of mainstream LED encapsulant polymers, and by adjusting the crosslinking density and doping concentration, the light scattering characteristics of the polymer matrix can be accurately simulated. From an industrial application perspective, devices fabricated with this model exhibit light emission characteristics comparable to commercial LED products under blue light excitation, and the color temperature can be precisely controlled by adjusting the phosphor particle size.

### 2.2. Slot Die Coating Observation Experiment

**Figure 2:**
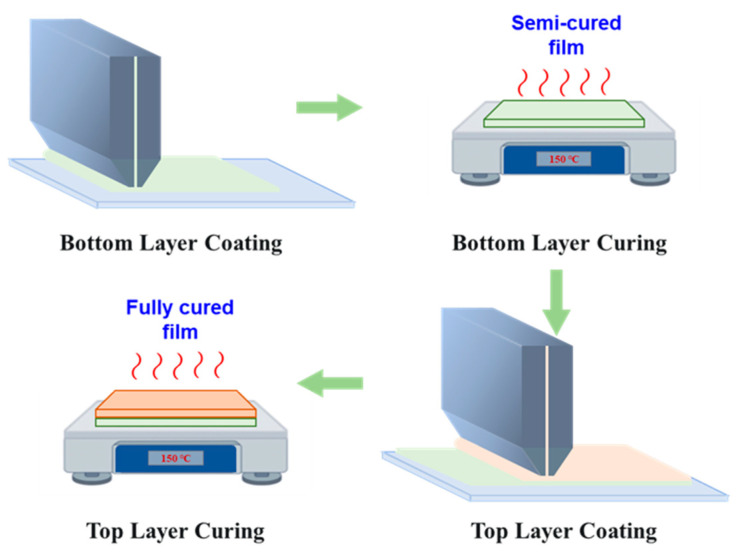
Schematic of the slot die coating observation experimental setup.

High-speed imaging was used to capture the real-time dynamics of liquid deposition onto a moving stainless substrate during the coat–dry–coat process, enabling precise visualization of transient interfacial phenomena and fluid motion throughout the initial wetting and spreading stages. Prior to the coating process, the substrates underwent a rigorous cleaning and preparation procedure to ensure surface cleanliness and promote uniform coating adhesion. To minimize bubble-related defects often observed in multilayer films, a modified double-layer fabrication procedure introduced a controlled partial cure of the base layer before applying the top layer, thereby reducing interfacial air entrapment. The complete experimental workflow is depicted schematically in Figure 2.

The experimental setup was configured to systematically assess how variations in contact angle and curing duration affect air entrainment. High-resolution image processing and analysis techniques tracked droplet wetting behavior on substrates with differing degrees of cure. In conventional polymer multilayer fabrication, the coat–dry–coat sequence requires the full drying of each layer before the next application, often inducing interfacial instabilities and trapped air [33]. In contrast, the present approach incorporates a partial curing step immediately after bottom-layer deposition. The resulting semi-cured film exhibits improved wettability, thereby minimizing air entrainment during top-layer coating. A final drying stage then completes the cure of the multilayer assembly, producing a defect-free polymer film.

### 2.3. Slot Die Coating Simulation

**Figure 3:**
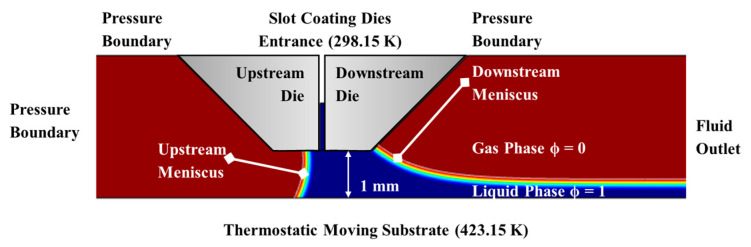
Coating flow structure in slot die coating processes.

A numerical model was developed with the finite element method in the COMSOL Multiphysics software 6.2 platform to investigate the fluid dynamics of the slot die coating process. A two-dimensional simulation was performed to analyze the flow behavior and interfacial evolution of the coating liquid during deposition, as illustrated in Figure 3. In the coating zone, a stable liquid meniscus forms between the slot die and the moving substrate. The simulation domain includes the slot coating die entrance, the upstream die, the downstream die, the pressure boundary, the fluid outlet, and the moving substrate. At the slot coating die entrance, a constant volumetric flow rate is imposed to mimic the fluid supply from the pump. The upstream and downstream dies are treated as no-slip solid boundaries, which constrain the fluid velocity to zero at the walls. The free surfaces exposed to air are treated as the pressure boundary to allow ambient pressure effects. At the fluid outlet, located at the end of the coating domain, a zero-pressure condition is applied to permit the continuous exit of liquid from the domain. The moving substrate at the bottom of the domain is modeled as a no-slip moving wall with a constant horizontal velocity corresponding to the coating speed. This stability is maintained through a delicate balance of viscous forces, fluid inertia, and pressure gradients. The leading and trailing edges of the coated liquid film are shaped into arc-like contours due to the combined influence of surface tension, gravitational forces, and interfacial curvature effects. For the purposes of the simulation, the coating fluid was assumed to behave as a Newtonian fluid. The process was modeled under transient and incompressible flow conditions to reflect realistic dynamic behavior during coating. To simplify the analysis and focus on liquid–substrate interactions, the effects of ambient airflow and solvent evaporation were not considered in this model.

The level set method was employed to track the evolution of the three-phase contact line, allowing for the accurate representation of interface deformation and dynamic wetting behavior. This method solves the coupled Navier–Stokes and Cahn–Hilliard equations, which govern fluid flow and phase interface dynamics, respectively. The fundamental governing equations and boundary conditions used in the simulation are detailed in references [34,35]:

(1) $$\frac{\partial \phi }{\partial t}+u\cdot \nabla \phi =\nabla \frac{3\gamma \sigma }{\sqrt{8}\varepsilon }\cdot \nabla \psi ,$$

(2) $$\rho \frac{\partial u}{\partial t}+\rho (u\cdot \nabla )u=\nabla \cdot [-pI+K]+F+\rho g,$$

(3) ρ∇⋅u=0,

where *t* is the simulation time, **u** is the velocity field, *σ* is the surface tension coefficient, *ɛ* is the parameter controlling interface thickness, *ρ* is the fluid density, *p* is the fluid pressure, and **F** is the sum of external forces acting on the fluid. Reinitialization parameter *γ*, phase-field auxiliary function *ψ,* and viscous stress tensor **K** are, respectively, defined as [34,35]

(4) $$\gamma =\chi \varepsilon^{2},$$

(5) ψ=−∇⋅ε2∇ϕ+(ϕ2−1)ϕ+8ε3σ∂f∂ϕ,

(6) $$K=\mu (\nabla u+{\left(\nabla u\right)}^{T})-\frac{2}{3}\mu (\nabla u)I,$$

where *χ* is the mobility parameter, *∂f/∂ϕ* is the dynamic viscosity, and *μ* is the dynamic viscosity.

The heat transfer part of fluid non-isothermal flow is mainly determined by the following governing equations:

(7) $$d_{z}\rho C_{p}\frac{\partial T}{\partial t}+\nabla \cdot q+d_{z}\rho C_{p}u\cdot \nabla T=d_{z}Q_{vd}+d_{z}Q+d_{z}Q_{p}+q_{0},$$

(8) $$Q_{vd}=\tau :\nabla u,$$

(9) $$q=-d_{z}k\nabla T,$$

where *Q_vd_* is the viscous dissipation, *τ* is the viscous tensor, *d_z_* is the thickness of the domain in the out-of-plane direction, *C_p_* is the specific heat capacity at constant stress, **q** is the heat flux by conduction, *k* is the fluid thermal conductivity, and *Q* contains additional heat sources.

In this study, *ϕ*_0_ = 0 in the gas phase and *ϕ*_1_ = 1 in the liquid phase. Initially, air fills the model, and the gas–liquid interface is located at the slot die coating entrance. The boundary conditions corresponding to the labeled area in Figure 3 are as follows.

Moving substrate [36]:

(10) $$u=\frac{u_{w}-(u_{w}\cdot n)n}{\left\|u_{w}-(u_{w}\cdot n)n\right\|}\left\|u_{w}\right\|,$$

(11) $$n\cdot \frac{3\gamma \sigma }{\sqrt{8}\varepsilon }\nabla \psi =0,$$

(12) $$n\cdot \varepsilon^{2}\cdot \nabla \phi =\varepsilon^{2}\cos(\theta_{w})\left|\nabla \phi \right|,$$

(13) $$T=T_{0},$$

Downstream die [36]:

(14) u=0,

(15) $$n\cdot \frac{3\gamma \sigma }{\sqrt{8}\varepsilon }\nabla \psi =0,$$

(16) $$n\cdot \varepsilon^{2}\cdot \nabla \phi =\varepsilon^{2}\cos(\theta_{w})\left|\nabla \phi \right|,$$

Upstream die [36]:

(17) u=0,

(18) $$n\cdot \frac{3\gamma \sigma }{\sqrt{8}\varepsilon }\nabla \psi =0,$$

(19) $$n\cdot \varepsilon^{2}\cdot \nabla \phi =\varepsilon^{2}\cos(\theta_{w})\left|\nabla \phi \right|,$$

Fluid outlet [36]:

(20) $$[-pI+K]n=-p_{0}n,$$

Slot coating die entrance [36]:

(21) $$u=U_{\mathrm{in}},$$

(22) $$\phi_{\mathrm{in}}=\phi_{1},$$

(23) $$-n\cdot q=d_{z}\rho ▵Hu\cdot n,$$

(24) $$\Delta H=\int_{Tset}^{T}C_{p}dT,$$

Pressure boundary [36]:

(25) $$[-pI+K]n=-p_{0}n$$

where **n** is the unit vector of the base system, *T*_0_ is the constraint temperature, *θ*_u_ is the static contact angle of the fluid with respect to the substrate material, *f*_0_ is the normal stress, **u**_w_ is the substrate movement velocity, *p*_0_ is the standard atmospheric pressure, and *T*_set_ is the substrate temperature.

### 2.4. Validation of Simulation Model

**Figure 4:**
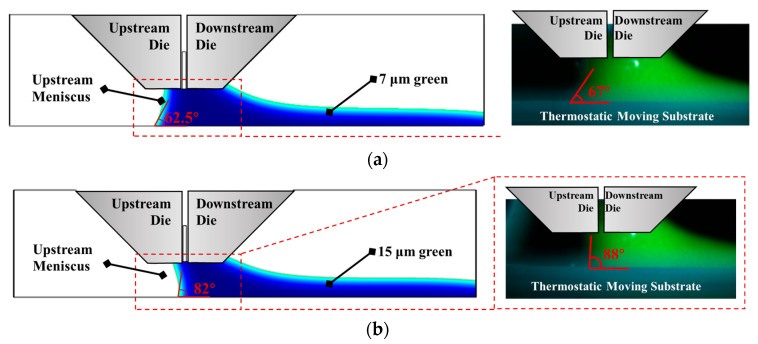
Comparison of the slot die coating process between experiments and simulations: (**a**) 7 μm green particles; (**b**) 15 μm green particles.

Before conducting simulations of the air entrainment phenomenon, a validation was performed to assess the influence of temperature-dependent viscosity and its effect on the contact angle during the slot die coating process. This preliminary simulation aimed to verify the accuracy and reliability of the developed numerical model by comparing the results with those reported in previous experimental and computational studies. To ensure a representative comparison, simulations were carried out using green phosphor particles with average diameters of 7 μm and 15 μm. The simulated meniscus profiles obtained under these conditions are presented on the left side of Figure 4, while the corresponding experimental images are shown on the right. The validation results demonstrate that the upstream and downstream meniscus shapes obtained from the simulation closely resemble those observed experimentally. The predicted contact angle values exhibit a maximum deviation of less than 6.8% from the measured values.

These findings confirm the robustness and predictive capability of the proposed slot die coating model. The validated model can therefore be confidently applied to investigate complex interfacial phenomena, including air entrainment mechanisms, under various operating conditions in the slot die coating process.

## 3. Results and Discussions

### 3.1. Air Entrainment Mechanism of Polymer Multilayer Films

The polymer multilayer film fabrication process via the coat–dry–coat technique was systematically investigated, with a particular focus on the occurrence of air entrainment during the top layer coating step. Through controlled experiments, it was observed that the formation of bubble defects is significantly more pronounced when the top polymer layer is applied onto the bottom layer, compared to the conventional single-layer coating process.

**Figure 5:**
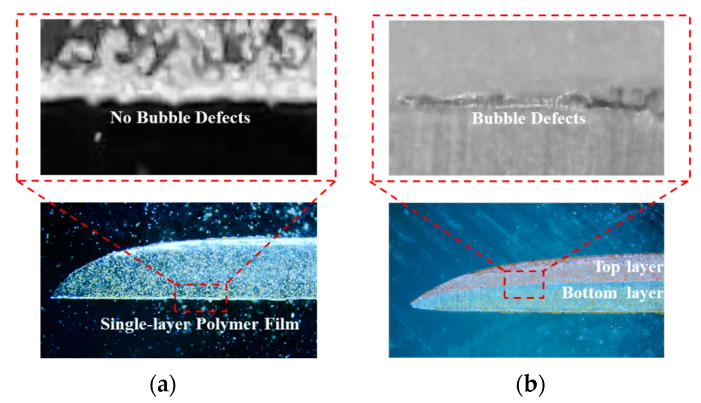
The comparison of single-layer and multilayer polymer film preparations. (**a**) Single-layer polymer film preparation; (**b**) multilayer polymer film preparation.

As illustrated in Figure 5a, the single-layer polymer film exhibited a uniform interface without any visible bubble defects between the film and the substrate. In contrast, the multilayer coating process resulted in noticeable bubble effects at the interface between the top and bottom polymer layers, as shown in Figure 5b. This interfacial air entrainment is likely caused by incomplete wetting or unfavorable flow dynamics during the top layer coating step. This result was obtained under identical coating conditions, including the coating speed, coating gap, slot gap, and ambient environment. These findings highlight a critical challenge in the multilayer film fabrication process and underscore the importance of optimizing coating conditions to suppress air entrainment and improve overall film quality.

**Figure 6:**
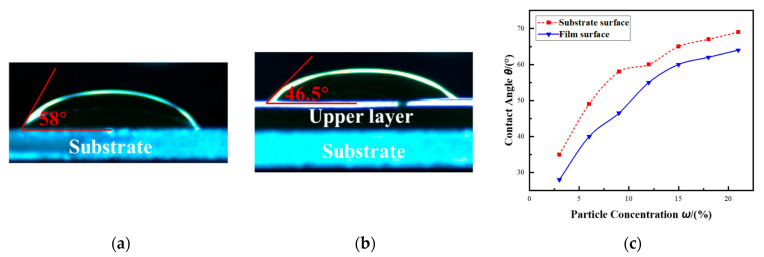
(**a**) A comparison of the measured static contact angle on the bare substrate and the cured bottom layer. (**b**) The variation in contact angles on the bare substrate and cured bottom layer with increasing particle concentration. (**c**) The effect of particle concentration on contact angle.

In order to investigate the cause of air entrainment during the multilayer polymer film coating process, contact angle measurements were systematically conducted using the sessile drop method on both the bare substrate and the cured bottom film surface. The sessile drop method is used to determine the contact angle between a liquid droplet and a solid surface by analyzing the three-phase contact line formed at their interface. In this study, a precision droplet generation system was employed to deposit droplets from a height of 2 mm onto the solid substrate. The droplets were allowed to rest on the surface for 3 s to ensure the formation of a stable three-phase contact line. Subsequently, the droplet shape was observed using an optical microscope, and the base diameter of the droplet was measured with a calibrated scale, enabling accurate calculation of the contact angle. As shown in Figure 6a, the measured static contact angle on the bare substrate was 58°, while the contact angle on the top surface of the cured bottom layer was significantly lower at 46.5°. These results were obtained under identical ambient conditions and polymer solution properties, ensuring a valid comparison of surface wettability. These measurements were conducted under the environmental temperature of 25 °C, using a polymer solution with a fluorescent particle concentration of 10%, thereby ensuring a valid and consistent comparison of surface wettability between the substrate and the cured film surface. This noticeable difference in contact angle reflects the differences in surface energy between the two interfaces. The higher contact angle measured on the substrate indicates greater liquid–solid interfacial tension and relatively weaker adhesion forces at the three-phase contact line, while the lower contact angle on the cured film surface suggests improved surface wettability and stronger affinity between the liquid and the solid.

However, enhanced wettability, as indicated by a lower contact angle, does not always ensure no defect polymer film formation. During the multilayer coating, a low contact angle at the interface can lead to the asymmetric spreading of the fluid across the cured bottom layer. This condition promotes instability in the upstream meniscus, where the fluid becomes sensitive to perturbations due to insufficient resistance at the contact line. As a result, the upstream meniscus may advance unevenly, disrupting the stable displacement of air and preventing the air entrainment. Therefore, under dynamic coating conditions, a smaller contact angle may increase the likelihood of air entrainment due to unstable upstream meniscus dynamics that fail to maintain continuous air displacement during the coating process.

Furthermore, Figure 6c presents contact angle measurements conducted on both the bare substrate surface and the surface of the fully cured polymer film, under varying concentrations of fluorescent particles dispersed in the polymer solution. The results show two consistent trends. First, for a given particle concentration, the contact angle measured on the substrate surface is consistently higher than that on the film surface, indicating that the substrate exhibits superior wettability. Second, and more notably, the contact angle on both surfaces increases progressively as the concentration of fluorescent particles in the solution increases.

This increase in contact angle with particle concentration can be attributed to several interrelated mechanisms. As the particle concentration increases, the particle–liquid and particle–solid interactions become more influential in governing wetting behavior. The fluorescent particles used in the suspension interact only weakly with the polymer matrix, disrupting cohesive forces within the liquid and reducing its affinity for the substrate. This leads to a higher contact angle. Moreover, greater particle concentrations increase the suspension’s effective viscosity, hindering liquid spreading. The resulting resistance to flow slows the wetting process and causes the droplet to retain a more rounded shape, further elevating the apparent contact angle.

**Figure 7:**
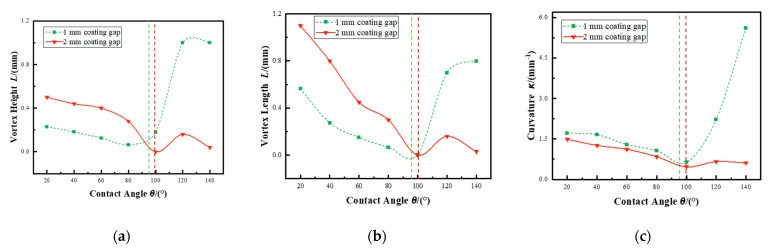
(**a**) The effect of contact angle on vortex height. (**b**) The effect of contact angle on vortex length. (**c**) The effect of contact angle on upstream meniscus curvature.

To further investigate the influence of surface wettability in air entrainment during the fabrication of multilayer polymer films, the numerical simulations were conducted focusing on the influence of contact angle. Previous studies by Fang et al. have shown that air entrainment is closely associated with vortex formation near the upstream meniscus and the contact region with the substrate [20]. In the present study, the morphology of these vortices, defined by their height and length, was used as an indicator of the intensity of air entrainment. Figure 7a,b illustrate the correlation between contact angle and vortex morphology under varying coating conditions. Figure 7c illustrates the variation in the curvature of the upstream meniscus as a function of the contact angle. As the contact angle changes, noticeable alterations in the meniscus profile are observed, reflecting the sensitivity of the fluid interface shape to surface wettability conditions.

In slot die coating processes with a coating gap of 1 mm, where inertial forces are predominant, the contact angle significantly influences the curvature of the upstream meniscus, thereby affecting vortex formation and the potential for air entrainment. Under these conditions, a gradual decrease in vortex height and length was observed as the contact angle increased up to approximately 95°, indicating a reduction in vortex formation intensity with increasing wettability. As the contact angle increases, the curvature of the upstream meniscus decreases significantly, resulting in a flatter liquid–air upstream meniscus. This change directly reduces the capillary pressure gradient across the meniscus, thereby weakening the driving force for vortex formation in the coating bead. This suppression of vortex formation effectively decreases the probability of air entrainment into the coating layer. However, beyond this threshold, both vortex height and vortex length increased sharply. As the contact angle increases beyond this threshold, the upstream meniscus transitions from a relatively flat profile to a more pronounced curvature. This change enhances the capillary pressure gradient across the meniscus, intensifying the local flow instabilities at the upstream meniscus. The increased curvature facilitates the formation of larger vortices due to the stronger interplay between inertial forces and surface tension effects. These larger vortices have a greater capacity to trap air, resulting in an increased likelihood of air entrainment within the coating layer.

When the coating gap was increased to 2 mm, the viscous forces become predominant over inertial effects, and the influence of the contact angle on the upstream meniscus curvature and flow behavior exhibits distinct characteristics compared to the inertial-dominated mechanism. As the contact angle increases up to approximately 100°, a gradual decrease in both vortex height and vortex length is observed, indicating that enhanced wettability suppresses vortex formation under viscous-dominated conditions. This suppression is attributed to the reduced curvature of the upstream meniscus, which flattens progressively as the contact angle rises. However, once the contact angle exceeds the threshold of 100°, a slight increase in vortex size is noted, followed by a subsequent decrease with further increases in contact angle. Also, the upstream meniscus curvature increases slightly before declining again. The brief resurgence in curvature and vortex intensity beyond the threshold may be attributed to a transitional regime where surface tension effects momentarily reinforce localized flow disturbances despite the dominance of viscous damping. As the contact angle continues to rise, the upstream meniscus stabilizes once more, with viscous forces effectively dissipating perturbations and reducing vortex formation.

### 3.2. Preparation of Bilayer Polymer Films Using the Semi-Cured Method

To suppress the air entrainment during the polymer multilayer film fabrication process, this study employed the semi-cured “coat–dry–coat” process, as introduced in Section 2.2. In this approach, the bottom layer is subjected to a controlled partial curing process prior to the coating of the top layer. Such a transitional surface exhibits modified wettability characteristics, which play a crucial role in regulating the interfacial dynamics during the subsequent top layer coating. By adjusting the curing degree of the bottom layer, it becomes possible to optimize surface properties that enhance coating stability, suppress undesirable interfacial instabilities, and significantly reduce the air entrainment between layers.

**Figure 8:**
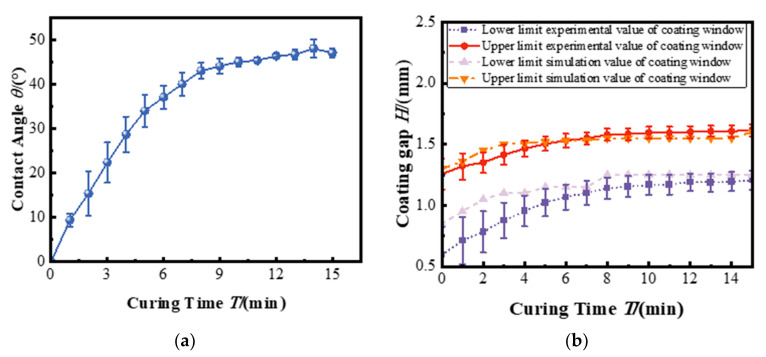
(**a**) Effect of curing time on contact angle. (**b**) Polymer multilayer coating window under different curing times.

Figure 8a illustrates the evolution of the top surface contact angle of the bottom layer as a function of curing time. It is observed that the contact angle increases with longer curing time. This trend reflects the surface gradually transforming from a wettable, low-viscosity liquid to a less wettable, semi-solid interface. As the curing progresses, molecular mobility is reduced and surface energy is altered, making the surface increasingly resistant to wetting by the top layer coating solution. Figure 8b illustrates the maximum and minimum coating gaps required to maintain no defect top-layer coating on the semi-cured bottom films for the different curing times. These two limits define the coating process boundaries, beyond which the air entrainment is likely to occur. With increasing curing time, both the upper and lower bounds of the coating gap expand, reflecting the enhanced resistance of the bottom layer to interfacial deformation as it transitions from a fluid to a semi-solid state. At shorter curing times, the bottom layer remains more fluidic, providing favorable wetting conditions and the ability to adapt to interfacial stresses. The relatively high wettability suppresses the formation of clockwise vortices under large coating gaps, allowing the coating bead to remain stable over a broad range of gap values. This results in a wide effective coating window, defined as the difference between the maximum and minimum allowable coating gaps. As curing time exceeds approximately 9 min, the bottom film becomes significantly less wettable and more rigid. Although the absolute gap limits continue to rise, the effective coating window narrows. This is because the low-wettability surface amplifies the flow instabilities, especially at the low coating gaps, increasing the vortex growth and air entrainment.

Figure 8b indicates that the upper limit of the coating window predicted by the simulation shows good agreement with the experimental data. However, a notable deviation is observed at the lower limit, where the simulation consistently overestimates the minimum feasible coating gap compared to experimental measurements. This mismatch is primarily attributed to the simplifications adopted in the initial simulation model, which assumes a constant fluid viscosity and fails to account for the temperature-dependent viscosity variations that arise during the partial curing process of the bottom polymer layer.

**Figure 9:**
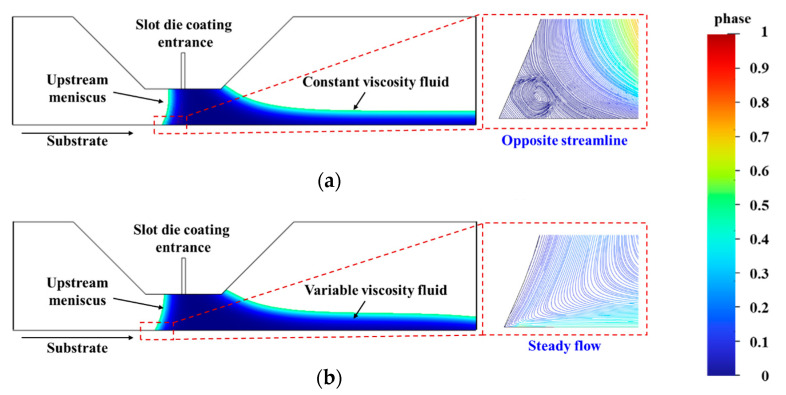
Slot die coating simulation results. (**a**) Constant viscosity. (**b**) Considering the effect of temperature on viscosity.

To resolve this discrepancy, an improved simulation was conducted by incorporating the temperature-dependent variation in fluid viscosity. The results demonstrated a significant change in the flow pattern at the upstream meniscus–substrate interface. In the initial model with constant viscosity, as illustrated in Figure 9a, opposite streamlines were observed near the upstream contact line, indicating the formation of vortices. These vortices can promote air entrainment and undermine the stability of the coating process. However, upon incorporating the temperature-dependent viscosity into the simulation model, a significant alteration in the flow behavior near the upstream meniscus was observed. As illustrated in Figure 9b, the previously present reverse flow region was eliminated, and the streamlines became steadier.

**Figure 10:**
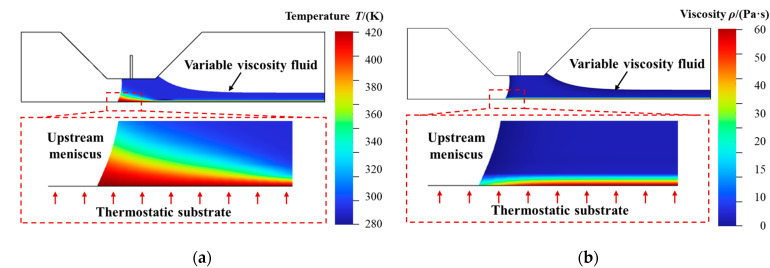
Slot die coating simulation results. (**a**) Temperature distribution. (**b**) Viscosity distribution.

To further investigate the impact of temperature on the viscosity distribution and the resulting flow behavior during the slot die coating process, the simulation results shown in Figure 10a,b were analyzed. Figure 10a illustrates the temperature distribution, while Figure 10b presents the viscosity distribution in the coating process. As shown in Figure 10a, a significant temperature increase is observed between the upstream meniscus and the bottom film surface. Additionally, the temperature on the bottom film surface is relatively high, suggesting that this area of the top film is undergoing partial curing, which is often characterized by a rise in viscosity. This temperature-induced change in viscosity is a key factor that contributes to the formation of a solidified film.

The viscosity distribution shown in Figure 10b further demonstrates a clear viscosity gradient in the contact area between the upstream meniscus and the bottom film surface. The gradient emerges due to the spatial variation in temperature, which accelerates crosslinking kinetics non-uniformly across the film. Also, as the temperature rises in these areas, the viscosity increases, which leads to the solidification phenomenon. An increase in viscosity is observed at the bottom of the top film relative to the surrounding areas, thereby significantly diminishing the local flowability. This phenomenon is consistent with the results in the previous simulations, where the flow at the upstream meniscus and the substrate contact region was found to be stable once the effect of temperature on viscosity was taken into account.

**Figure 11:**
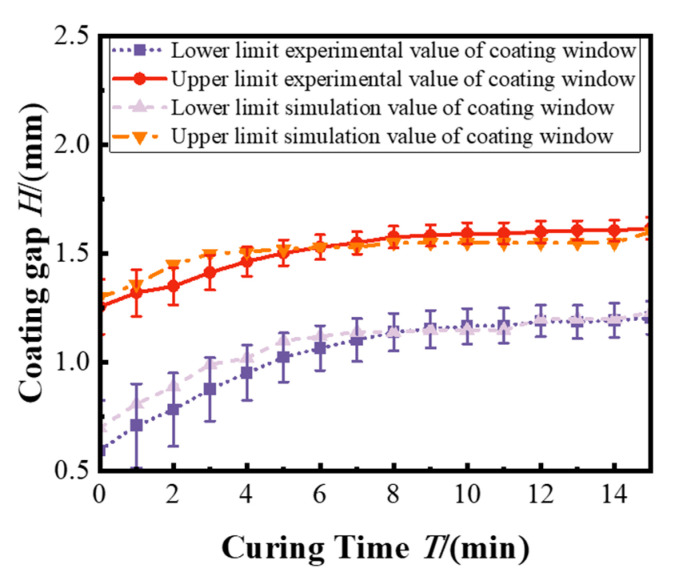
Coating window considering the temperature dependence of viscosity.

Figure 11 illustrates the updated coating window, which establishes a quantitative correlation between the curing time and the limit coating gap. In contrast to the previous constant viscosity model, the revised model accounts for the dependence of polymer viscosity on curing temperature. This factor plays a critical role in flow stability and polymer film formation. As a result, the simulated coating window matches much more closely with experimental observations, reflecting the improvement in the model. As shown in Figure 11, the limit of the coating window is more accurately predicted when the temperature dependence of viscosity is taken into account. Notably, the lower limits of the coating window, which was previously overestimated in the simulations, now show close agreement with the experimental data.

Quantitatively, the overall error between the simulation and experimental data for limit coating gap prediction decreased from approximately 11.49% in the constant-viscosity model to 4.99% in the temperature-dependent model. This improvement validates the inclusion of temperature–viscosity coupling that captures localized flow dynamics near the upstream meniscus and substrate interface, which are critical in determining the vortex formation and air entrainment.

## 4. Conclusions

This study systematically examined the mechanisms of air entrainment during multilayer polymer film fabrication using a combination of experimental observations and numerical simulations. The results highlight the key roles of surface wettability, contact angle variations, and temperature-dependent viscosity in governing interfacial stability and the formation of bubble defects. To address these issues, a semi-cured slot-die coating approach was introduced and demonstrated to be effective in reducing air entrapment and broadening the stable coating window for multilayer applications. The main conclusions of this study are as follows:

- The experimental results demonstrated that air entrainment is more likely to occur during the top-layer coating of polymer multilayer films compared to single-layer coatings. This phenomenon is due to the contact angle differences between the substrate and the cured bottom film, where an enhanced wettability of the lower layer can lead to asymmetric spreading and unstable upstream meniscus dynamics, ultimately promoting air entrainment.
- Numerical simulations revealed that contact angle plays a critical role in controlling vortex formation and meniscus curvature under both inertial- and viscous-dominated coating conditions. A threshold behavior was observed: moderate increases in contact angle reduced vortex intensity and helped suppress air entrainment. However, when the contact angle exceeded a certain limit, the resulting increase in meniscus curvature intensified flow instabilities and enlarged vortex structures, ultimately promoting greater air entrapment.
- Introducing a semi-cured bottom film in the coat–dry–coat process enabled the precise tuning of surface wettability, thereby enhancing coating stability. Additionally, incorporating temperature-dependent viscosity into the simulation model markedly improved the accuracy of coating window predictions, reducing the coating gap error from 11.49% to 4.99%. These results underscore the critical role of temperature effects on viscosity gradients in accurately modeling multilayer coating dynamics.

## Figures and Tables

**Table 1:** Parameters of silica gel and particles.

Property	Unit	Value
Silica gel viscosity (ηF) (25 °C)	Pa·s	6.0
Silica gel density (*ρ*_F_)	kg/m^3^	1020
Particle composition	/	SrAl_2_O_4_: Eu, Dy
Particle density (*ρ*_P_)	kg/m^3^	4200
Surface tension	N/m	0.2

## Data Availability

The original contributions presented in this study are included in the article. Further inquiries can be directed at the corresponding author.
